# Young people’s mental health: an NHS primary care perspective, 10 years on

**DOI:** 10.3399/BJGP.2025.0743

**Published:** 2025-12-16

**Authors:** Daniel Romeu, Prathiba Chitsabesan, Faraz Mughal

**Affiliations:** 1 Leeds Institute of Health Sciences, University of Leeds, Leeds LS2 9NL, UK; 2 Pennine Care NHS Foundation Trust, Manchester OL6 7SR, UK; 3 School of Medicine, Keele University, Keele ST5 5BG, UK

In 2016, an editorial in the *BJGP* warned that Children and Young People’s (CYP) mental health services were the ‘Cinderella of Cinderella services’ and called for primary care to play a more pivotal role in early identification, intervention, and co-ordination of support.^1^ Since then, the scale of this issue has grown; referrals to specialist Child and Adolescent Mental Health Services (CAMHS) have increased, waiting times have lengthened, and general practice remains one of several universal settings, alongside schools, at the forefront of an under-resourced system struggling to meet demand^2^.

The policy landscape, however, has shifted significantly. Recent developments, including the 10-Year Health Plan^3^ and NHSE’s National Implementation Guidance for Urgent and Emergency CYP Mental Health Care^4^, signal a system moving towards integration, prevention, and earlier intervention. The most transformative change for primary care is the introduction of Neighbourhood Multidisciplinary Teams (NMDTs) for CYP, closely aligned with Neighbourhood Health Centres (NHCs) described in the 10-Year Health Plan, and the continued national roll-out of Mental Health Support Teams (MHSTs) in schools and colleges. Together, these initiatives shift mental health care closer to where young people live, learn and seek help.^5^

## Ten years later: shifting contexts and persistent gaps

The case for transformation is clear. Prevalence of CYP mental health problems has continued to rise: one in five young people in England are now estimated to experience a probable mental health disorder^5^ and around 48% of lifetime mental disorders begin by age 18 and 63% by 25^6^. Yet only about 40% of those affected receive treatment, leaving a substantial treatment gap^7^.

“...one in five young people in England are now estimated to experience a probable mental health disorder^5^ and around 48% of lifetime mental disorders begin by age 18...”

Rising prevalence reflects the complex interplay of social, economic and cultural factors, including poverty, income inequality, exposure to adversity and adverse childhood experiences (ACEs), and the digital environment. While increased awareness and stigma have partly driven the rise in referrals, genuine need has also grown. Research evidence on population-level harms of social media remains mixed, but there is a consensus that excessive screen time and exposure may exacerbate distress among vulnerable CYP.

Over the past decade, national strategies (‘No Health Without Mental Health’, ‘Future in Mind’, ‘The Five-Year Forward View for Mental Health’, and the ‘NHS Long-Term Plan’) have emphasised early intervention, parity of esteem, and investment in community treatment. The Long-Term Plan brought new funding and places health inequalities at the heart of reform.^8^ The THRIVE framework promoted a departure from the rigid, inflexible CAMHS tier system towards a needs-led approach^9^. Implementation remains uneven, and many families still struggle to access timely, appropriate support.

Crisis care has expanded beyond emergency departments (EDs): while CYP mental health presentations comprise only around 2.5–4.5% of all ED attendances^10^, most crisis contacts now occur through helplines, home treatment teams, and voluntary sector services. Alongside the growth of MHSTs, community eating disorder services, and 24/7 crisis care, this reflects progress towards three shifts: from hospital to community, reactive to proactive, and treatment to prevention.

## Neighbourhood multidisciplinary teams (NMDTs): integrating care around families

NMDTs represent the next major step in CYP mental health transformation. These teams – the CYP counterpart to the Neighbourhood Health Centres outlined in the 10-Year Health Plan^3^ – will operate in close partnership with Primary Care Networks (PCNs), linking GP teams with education, social care, and voluntary sector partners. Their purpose is to integrate care vertically and horizontally, bridging organisational boundaries to support prevention, early intervention, and equitable access.

NMDTs will work alongside MHSTs, health visitors, school nurses, Family Hubs, and voluntary and community partners. By bringing these networks together, NMDTs can help address fragmentation and ensure young people receive joined-up care before crisis occurs. Models such as Connecting Care for Children and The CHILD Model demonstrate that integrated paediatric-primary care collaboration can reduce ED presentations and inpatient admissions while improving clinician confidence and family experience.

“General practice remains the most common entry point for young people seeking help for mental health problems, and its strengths [...] are vital to building trust and therapeutic alliance.”

For GP clinicians, NMDTs offer tangible benefits: enhanced access to specialist input, higher referral acceptance and greater confidence in managing CYP mental health needs in primary care. They also strengthen primary care’s role in population health, enabling GPs to identify inequalities, target prevention and support whole-family approaches. Digital integration, including shared care plans and messaging through the NHS App, can improve continuity, although digital options should complement, not replace, in-person care.

NMDTs will not deliver crisis care directly, but work alongside crisis helplines, home treatment teams, eating disorder services, crisis cafés and day services to wrap support around young people and families. Examples such as the East Lancashire Child and Adolescent Service (ELCAS) and The Well Centre in London illustrate how co-location, shared data, and youth-friendly environments improve access and engagement.

## The critical role of primary care in CYP mental health

General practice remains the most common entry point for young people seeking help for mental health problems, and its strengths — cross-sectoral holistic frontline person-centred care — are vital to building trust and therapeutic alliance. Epidemiological data show that CYP presenting to general practice are twice as likely to have a mental health problem.^11^ If young people see a named professional over time, then rapport develops and repeated retelling of traumatic stories is avoided, because relational continuity creates psychological safety.^12^

However, general practice faces substantial workload pressures and an acute workforce crisis. Several changes are needed to equip teams. Training and staff development should be prioritised, and CAMHS training placements should be a core component of GP training. Embedded roles are key to sustainability; CYP mental health practitioners should be integrated within practices to support mental health assessments, risk management, and brief, targeted interventions. GP clinicians should continue to prioritise sensitive enquiry of mental health needs, because one well-timed question can open the door to disclosure.^13^

## Linking crisis with continuity

Primary care services and NMDTs have an upstream role in identifying young people at risk of crisis: for example, through shared data platforms integrating GP, CAMHS, and education records, or regular MDT case discussions focused on self-harm, not in education, employment, or training (NEET) status or looked-after-child indicators. National standards for CYP crisis mental health care describe four core functions of crisis services: single point of access via NHS 111 to rapid mental health triage, biopsychosocial assessments in EDs and community settings, availability of brief responses and interventions, and intensive home treatment as an alternative to hospital admission.^4^ Neighbourhood teams complement these by maintaining engagement, supporting transitions, and helping families navigate services after a crisis episode.

GP teams can support continuity after crisis by maintaining contact and contributing to transition planning where appropriately resourced, particularly for young people moving between CAMHS and adult services, while crisis and home treatment teams usually lead immediate post-crisis service provision. If discharging teams make structured linkage calls that include GP and community services, then motivation towards recovery and engagement with services is likely to increase, because young people perceive care as extending beyond crisis and addressing underlying distress.^14^

## Implications for practice and policy

Meeting the growing mental health needs of young people requires system-level and clinical action. Several priorities emerge:

1. **Invest in neighbourhood care**: connect primary care, MHSTs, social care, and voluntary neighbours at neighbourhood level, with shared workforce and leadership.
2. **Right care, right time**: deliver needs-based care that emphasises prevention and early intervention as well as crisis response, ensuring access to the right support at the right time.
3. **Address inequalities**: prioritise roll-out of neighbourhood teams and new pathways in areas of greatest deprivation and among groups experiencing health inequalities.
4. **Evaluate meaningfully**: monitor access, waiting times, experience, and outcomes that reflect what young people and families value. Lower activity may signal disengagement, not success.
5. **Support data and digital integration**: link records across health, education, and social care systems to enable population-based planning and proactive identification, while ensuring equitable hybrid access for those who prefer face-to-face contact.
6. **In partnership**: co-design and co-evaluate services to build trust, responsiveness, and lasting engagement.

## Call to action

In 2016, the *BJGP* called for better training, innovation, and integration to strengthen CYP mental health.^1^ In 2026, the evidence is clearer, the urgency greater, and the infrastructure stronger. With sustained investment and consistent implementation, the next decade could transform CYP mental health from the ‘Cinderella of Cinderella services’ into a model of integrated, prevention-focused, community-based care.
